# Intrinsic phenotypic stability of a bi-stable auto regulatory gene

**DOI:** 10.1038/srep22951

**Published:** 2016-03-10

**Authors:** Azim-Berdy Besya, Andreas Grönlund

**Affiliations:** 1 Umeå Plant Science Centre, Department of Plant Physiology, Umeå University, SE-901 87 Umeå, Sweden

## Abstract

Even under homogenous conditions clonal cells can assume different distinct states for generations to follow, also known as epigenetic inheritance. Such long periods of different phenotypic states can be formed due to the existence of more than one stable state in the molecule concentration, where the different states are explored through molecular fluctuations. By formulating a single reaction variable representing the birth and death of molecules, including transcription, translation and decay, we calculate the escape time from the phenotypic states attained from autocatalytic synthesis through a Fokker- Planck formulation and integration of an effective pseudo-potential. We calculate the stability of the phenotypic states both for cooperative binding feedback and dimer binding feedback, resulting in non-linear decay.

The cellular machinery often work with few molecules at hand. A gene may exist as one single copy on the DNA and the copy number of the messenger RNA and following protein are typically very low^1^. Chemical reactions are probabilistic – before a reaction can take place the participating molecules first need to find each other through random motion, chemical bonds form at random. Molecular decay is also random. Combining low copy number and random birth and death of molecules make biochemical reaction networks noisy, even if we eliminate all external variations in the environment of a given biochemical process. Two clonal cells placed in the same environment may therefore at times exhibit very different behaviour. Fluctuations generated from birth and death of molecules in a reaction network are commonly referred to as *intrinsic fluctuations* whereas *extrinsic fluctuations* are fluctuations of the environment that the studied reaction network are embedded in, i.e. variations in the reaction rates of the reaction network^2^,^3^. The magnitude of the molecular fluctuations can be altered by a change in the reaction rates and by regulatory processes. Negative autoregulation is shown both to reduce noise^4^ and to amplify fluctuations if the feedback mechanism is delayed^5^,^6^,^7^ or if the feedback is strong^8^. The fidelity of regulatory control is also limited by the molecular noise associated with the process of transmitting biochemical signals^9^ and by finding the regulatory sites^10^,^11^,^12^. However, there are no a priori reasons to assume molecular fluctuations only being detrimental for an organism. In theory noise could be exploited for enhancing the sensitivity of regulatory control processes^13^. Moreover, there are many situations where noise is an essential component of a process. Overcoming potential barriers by thermal fluctuations are what drives chemical reactions in the first place and in are this respect of course essential, but potential wells and multi-stable states can also be formed by the dynamics of molecule synthesis, e.g. if the synthesis is enhanced by its own presence. The consequence of such feedback is that there are two or more stable concentrations that the different molecule species of the reaction network may attain. Autocatalytic protein synthesis is shown both theoretically and experimentally to generate meta stable states and the consequence of such seemingly simple positive feedback is that genetically identical individuals may even at identical homogenous conditions attain different phenotypic states^14^,^15^,^16^,^17^,^18^. The states are referred to as phenotypic states, since the protein concentration may increase (or decrease) by orders of magnitude and last for generations. E.g. the spontaneous exit of the lysogenic state for the lambda phage is only about 10^−7^–10^−5^ per cell and generation^18^,^19^ showing that dynamic stability can be on par with the stability of DNA^20^,^21^. External perturbations, e.g. by sudden changes in the reaction rates, can push the biochemical reaction network from one metastable state to another. Random switching from one stable state to another may occur from extrinsic fluctuation or perturbations but also as a consequence of the intrinsic noise generated from the birth and death of molecules, without any external signal preceding the escape of the state. Random switching is exploited in bacteria as a stochastic survival strategy to blindly anticipate variations in the environment, where different rates of switching is resulting in different fitness^22^. Note that the random switching mechanism display an intrinsic time scale which is not triggered by changes in the environment. Random switching is also observed to induce antibiotic resistance in otherwise identical cells^23^.

Traditionally, calculations of the stability of metastable states are done for Brownian particles performing random walks in a potential well, or similar systems, where the noise is not a function of the position in space. For autocatalytic transcription factors, where metastable states in the concentration can be generated as a result of the protein exhibiting positive feedback on its own synthesis, the birth (and death) of molecules also generates fluctuations. Thus, not only is the potential a function of the protein concentration but also the noise, which makes analytical progress challenging. The analogue of such noise for a brownian particle escaping a potential well would be to have noise that is different at different locations in space. In addition, cellular molecules typically are synthesised in a series of steps where each intermediate product or state leading to the final product will also leave its contribution to the overall fluctuations. To account for the problem just described, the WKB method can be exploited to give approximate solutions in leading order up to a constant pre-exponential factor of the escape time^24^,^25^,^26^,^27^. We will derive another approximate method to account for the molecule dependent noise by taking the classic approach of integrating the Fokker-Planck equation^28^ where the pre-exponential factor directly will be given from the kinetic parameters. We will collapse the dynamics of all chemical components into a single variable and describe the noise level of the collapsed variable for concentrations outside of stable states such that the escape time can be integrated through a pseudo-potential. The calculations improves the accuracy of what can be obtained from a constant noise approximation and in addition accounts for intermediate product noise and non-linear degradation as a result of dimer formation.

## Results

The necessary criteria for generating two stable phenotypic states from positive transcriptional feedback is that the feedback is sufficiently non-linear, which can be quantified by the Hill coefficient^29^,^30^,^31^. We will restrict the calculations to feedback generating a Hill coefficient of two, the smallest coefficient that generates bi-stability. This can be achieved when monomers bind cooperative on the promoter to two adjacent binding sites or when proteins form dimers that bind on a single binding site.

**Figure 1:**
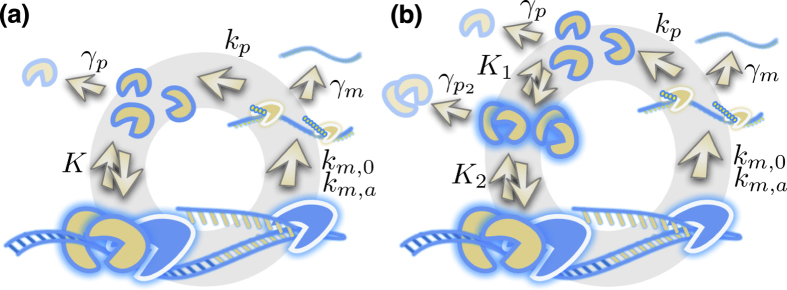
Illustration of the regulatory loop of the two studied motifs of positive autoregulation. The illustration display synthesis of messenger RNA from DNA, synthesis of proteins from messenger RNA and binding of the protein to the promoter to its own gene. The non-linearity of the feedback regulation is the same for the two studied motifs but is in (**a**) achieved by a cooperative binding of two proteins to the promoter and in (**b**) by forming dimers before binding to the promoter. An important difference, which is illustrated in (**b**), is that feedback mediated by dimers also imply that proteins decay not only as monomers but also as dimers. Whenever the promoter is occupied by proteins, and active, the synthesis rate of messenger RNA is boosted to *k*_*m*,0_ + *k*_*m*,*a*_ from the basal rate *k*_*m*,0_. Protein translation is proceeding with the rate *k*_*p*_. The dissociation binding constants between protein monomers and dimers *K*_1_ and the promoter *K*_2_ (dimers) and *K* (monomers). Decay rate constants of messenger RNA *γ*_*m*_, proteins *γ*_*p*_ and protein dimers 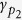.

The biochemical reaction schemes are illustrated in Fig. 1. In Fig. 1a,b cooperative binding feedback and dimer binding feedback is illustrated, respectively. Protein synthesis is modeled as a two-step process with mRNA as intermediate species. The gene can be either in it basal or activated state, active whenever the promoter is bound with protein, with mRNA synthesis rates of *k*_*m*,0_ in the un-bound state and *k*_*m*,0_ + *k*_*m*,*a*_ in the active state. The concentrations of; mRNA *m*, proteins *p* and protein dimers *p*_2_ decay with rates *γ*_*m*_, *γ*_*p*_ and 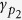, respectively. The autocatalytic positive feedback loop is then a result by the protein enhancing its own synthesis by binding to its promoter either in the form of dimers or by two proteins binding cooperatively.

We will derive a stochastic differential equation that describes the evolution of the feedback system through a single reaction variable *x*,


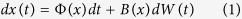


The equation contains a concentration drift term Φ(*x*) that is given by the (ensemble) average rate difference of synthesis and decay and a noise term *B*(*x*), originating from the small perturbations of birth and death of molecules. The drift term is calculated in the limit of large molecules whereas the noise term is given by the relative perturbation at finite molecule numbers. When we have expressed Φ(*x*) and *B*(*x*) we can through a pseudo potential, 
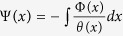
, where 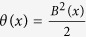, calculate the average escape time as


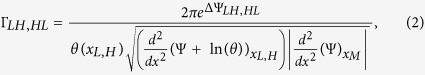


where ΔΨ_*LH*,*HL*_ = Ψ(*x*_*M*_) − Ψ(*x*_*L*,*H*_). The indices *L* and *H* denotes Low and High stable concentration which are separated by the potential peak at the Middle unstable point, *M*. The derivation is given in the Methods Section. Thus we need to choose our variable *x*, calculate Φ(*x*) and *θ*(*x*) and then integrate the pseudo potential Ψ(*x*) and insert into equation 2.

### Concentration drift and stability

The concentrations of the molecules illustrated in Fig. 1 will in the large molecule limit (for dimer binding feedback) evolve according to the following set of ordinary differential equations (ODE)


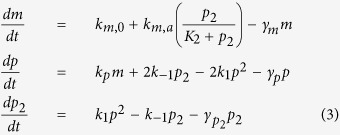


**Figure 2:**
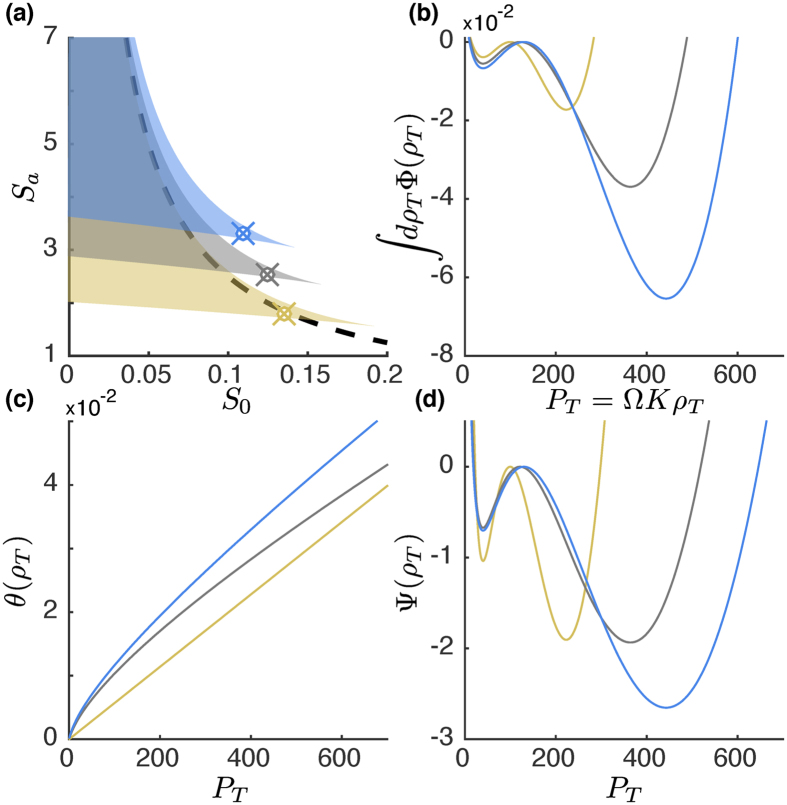
The potential and noise properties as a function of the total protein number *P*_*T*_ = Ω*Kρ*_*T*_. (**a**) The region of bi-stability for cooperative binding feedback (yellow) and dimer binding feedback with the decay rate parameter *α* = 0.5 (grey) and *α* = 1 (blue). The dashed line is the asymptote *S*_0_ = 1/4*S*_*a*_ gives the upper limit of the basal synthesis rate *S*_0_ generating bi-stability in the limit of large activation synthesis *S*_*a*_. (**b**) The potential, which is given by the integral of the concentration drift, of the three points marked in (**a**) as a function of the number of proteins. In (**c**) the noise *θ*(*ρ*_*T*_) needed for calculating the effective quasi-potential 
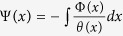
, de-pictured in (**d**).

Typically, the protein life-time is large compared to the mRNA life-time and the equilibration of monomer and dimer concentrations. Under such conditions we can formulate an equation for the concentration drift Φ(*ρ*_*T*_) in the total scaled protein concentration, *ρ*_*T*_ = *ρ* + 2*ρ*_2_ where 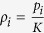 as


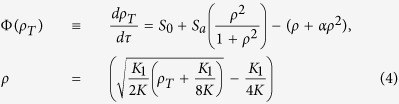


giving us the effective synthesis rate constants 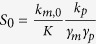, 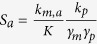 and the scaled dimer decay rate constant 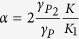 and the dissociation constants *K*^2^ = *K*_1_*K*_2_. Time is normalized by the protein life-time as *τ* = *γ*_*p*_*t*. The equations describing cooperative binding feedback, illustrated in Fig. 1a, can be obtained by letting *K*_1_ → ∞ making *α* = 0. Moreover, since *ρ*_*T*_ = *ρ* + 2*ρ*_2_ and *ρ*^2^ = *ρ*_2_*K*_1_/*K* we can formulate equation 4 closed in terms of any of the concentrations *ρ*, *ρ*_2_ or *ρ*_*T*_. For the rate parameters where the system displays bi-stability there are always two stable stationary solutions separated by an unstable one and the region of bi-stability is given by the interval (ρ_*,1_, ρ_*,2_) where both endpoints are solutions of Φ(ρ_*_) = 0 and Φ′(ρ_*_) = 0. The region of bi-stability is given by


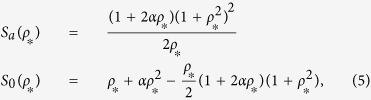


where ρ_*_ are the monomer concentrations separating the bi-stable region. The region of bi-stability is displayed in Fig. 2a. In the limit of *α* → 0 the dimer binding feedback give the same region of bi-stability as cooperative binding. In the limit of having large (scaled) activated synthesis rate, *S*_*a*_, the limit for having bi-stability is given by *S*_0_ = 1/4*S*_*a*_. Thus a large feedback activation need to be accompanied by a low basal synthesis rate for bi-stability to occur. In Fig. 2b we display the potential function, given by the primitive function of the concentration drift Φ(*ρ*_*T*_), at the three marked points in Fig. 2a.

### Intrinsic noise

We will now derive an expression for the intrinsic noise of the total concentration of proteins, *B*(*p*_*T*_), assuming that mRNA turnover and protein dimerization equilibration are both fast compared to protein turnover. One species, total protein copy number, *P*_*T*_, and three reactions,


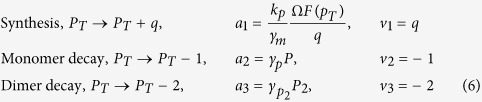


The function *F*(*p*_*T*_) in the propensity of the synthesis is given by the two first terms in the first row of equation 3, i.e the synthesis rate of proteins. However, we have scaled the propensity of synthesis with the average protein synthesised per mRNA and the stoichiometric correction *q*. The idea is that the average synthesis rate, which is the propensity times the stoichiometry, is not dependent on *q* and that *q* gives a measure of the burst rate of the protein synthesis in the full reaction system. Increasing *q* implies that more proteins are produced per synthesis event but that the synthesis events appear less frequently. W can calculate the variance *C* of the total protein concentration using the fluctuation dissipation relation (see Methods section), *C* = −*D*/2*A*, where


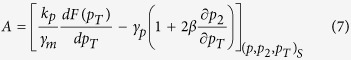


letting 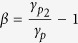 and where the diffusion component is


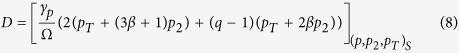


By matching the variance of the total protein concentration for the full system, calculated in the same way as above using fluctuation dissipation relation, we obtain the effective stoichiometry for the reduced system 6 to be *q* = 1 + 2*b* when mRNA decay is assumed to be rapid compared to protein decay (calculations of *q* is presented in the supplementary information). The parameter 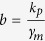 is the number of proteins translated per mRNA. We then obtain the noise component for the dimer binding feedback by inserting the calculated *q* in equation 8,


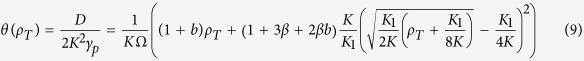


where we have re-written *ρ*_2_ in terms of *ρ*_*T*_. For cooperative binding feedback we get 
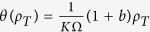
. Note that *θ*(*ρ*_*T*_) is only valid at the two stable fixed points *ρ*_*T*_ = *ρ*_*L*_ and *ρ*_*T*_ = *ρ*_*H*_, but we now assume that the dependence on *ρ*_*T*_ can be approximated to hold for all concentrations.

### Escaping the stable states

Now we know both Φ(*ρ*_*T*_) and *θ*(*ρ*_*T*_) are ready to calculate the escape time of the phenotypic states by integrating the quasi-potential Ψ(*ρ*_*T*_). The integration of Ψ is presented in the Supplementary Information and for brevity we just state the results here. For dimer binding feedback we obtain


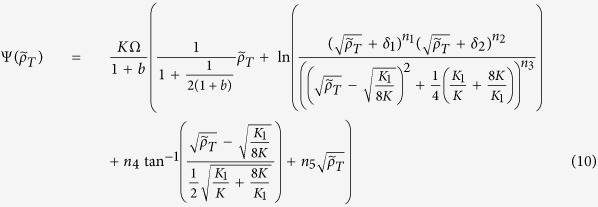


where 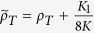. The parameters *δ*_*i*_ and *n*_*i*_ are presented in the Supplementary Information. For cooperative binding feedback


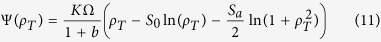


We note that the pseudo potential difference between the stable states and the unstable peak, ΔΨ_*LH*,*HL*_ = Ψ(*x*_*M*_) − Ψ(*x*_*L*,*H*_), scale to first order as 
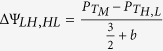
 for dimer binding feedback 
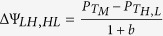
 for cooperative binding feedback.

**Figure 3:**
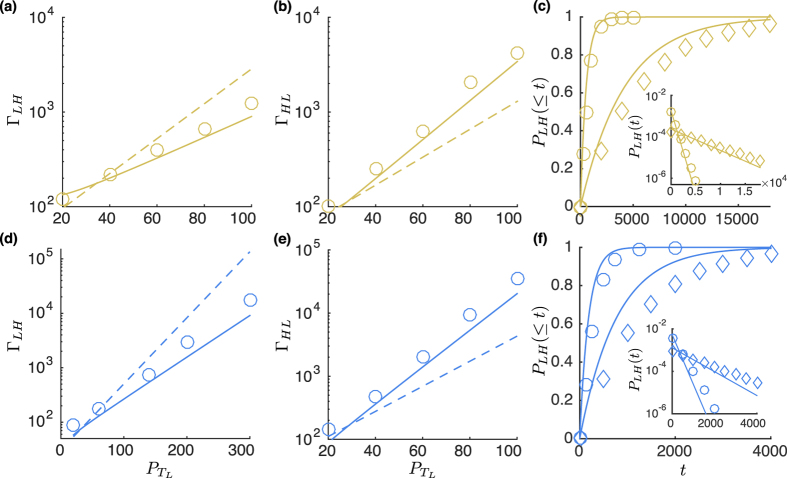
The accuracy of the escape time from the phenotypic low states *L* and high states *H* with increasing number of molecules. Upper panels show escape properties for cooperative feedback binding and lower panels for dimer binding feedback. The rate parameters are given from the yellow and blue points in Fig. 2a for cooperative and dimer feedback, respectively. In addition we set the monomer-to-dimer decay rate ratio to 10 for dimer binding feedback, making the lifetime of dimers ten times longer than monomers. Errors of the stochastic simulations are smaller than the symbols. The panels (**a**,**b**,**d**,**e**) show the mean escape rate, where *Γ*_*LH*_ denotes escape from Low to High state and vice versa. In the rightmost panels we display the probability of escaping the low state to the high state as a function of increasing time where the lines are the theoretical expected probability assuming an cumulative probability of the exponential distribution, 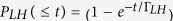. The small insets show the probability density function of escaping assuming an exponential distribution, 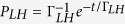. Errors are smaller that symbols and circles are for the low state having 80 proteins and diamonds with the low state having 160 molecules.

We have plotted both the noise component *θ* and the quasi potential Ψ in Fig. 2b,c. The escape time is obtained by insertion of Ψ(*ρ*_*T*_) and *θ*(*ρ*_*T*_) in equation 2 with *x* = *ρ*_*T*_. In Fig. 3a–c we display escape properties for cooperative binding feedback and in panels 3(d)–(f) for dimer binding feedback. The accuracy of the escape time is displayed in Fig. 3a,b,d,e where we as a reference also display the escape time calculated for the constant noise approximation (dashed line). The constant noise approximation is performed by approximating to the noise level at the stable concentration from which the escape is taking place, *θ*(*ρ*_*T*_) = *θ*(*ρ*_*L*_) or *θ*(*ρ*_*H*_)^32^. The benefit of accurately describing the noise is seen when comparing with the constant noise approximation, which is systematically underestimating the noise levels whenever going from the low to the high stable concentration, resulting in an escape time that is longer than what is obtained from exact stochastic simulations of the reaction network. For escaping from the high stable concentrations we see the opposite behaviour, which is natural since the noise is overestimated when approximated with the level at the high stable concentration. The stochastic simulations of the escape rate is done using Gillespie’s stochastic simulation algorithm^33^.

Now, since the fluctuations at the stationary points occur with a time-scale much shorter than the time scale of switching from one phenotypic state to another, implying that the actual jump from one phenotypic state to the another it fast compared to the time spent in either of the phenotypic states, it is reasonable to assume that the switching process is memoryless an can be approximated as a one-step process (similar to a regular chemical reaction). Under such assumptions the time between switching events can be described by the exponential distribution, with the average rate 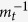 of switching as the only parameter describing the distribution. We test this assumption by measuring the probability of escaping from the low state to the high state during the time interval [0, *t*] from stochastic simulations (Figure 3c and 3f, diamonds and circles), that is the cumulative distribution *P*(≤*t*). The cumulative probability of the exponential distribution is given by 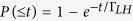, where we have inserted our calculated mean time of switching *m*_*t*_ = Γ_*LH*_. The small insets display the probability density function 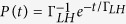.

## Discussion

We have presented a method for calculating the switching kinetics between phenotypic states, formed by autocatalytic synthesis, where the switching is driven by intrinsic fluctuations. The method relies on deriving a single reaction parameter by a separation of timescales and formulating an effective stoichiometric parameter such that both the concentration drift and fluctuations represent what is observed in the full system. Here we have applied the results on transcription factors binding to its own promoter and enhancing its own synthesis. Such feedback is the most common transcriptional feedback motif in bacteria^34^ with the possibility of generating meta-stable states. The feedback need to be sufficiently sensitive to generate metastable states and this can be achieved either by forming multimeric complexes before binding to the promoter or/and having multiple and cooperative binding sites on the promoter. Typically, transcription factors form multimeric complexes, as dimers or tetramers, and most likely it is not only due to improving the feedback sensitivity but also due to symmetry in the molecule’s binding to DNA. The formation of a multimeric complex of the feedback molecule give that the molecule can bind in several orientations which makes the search time to the specific binding site shorter, since the binding (and re-binding after dissociation) becomes more probable^35^. Assuming all other reactions parameters fixed, faster search kinetics of the transcription factor will increase the equilibrium binding constant. From the method in this paper, one may directly investigate such effects since both the pre-exponential factor and the exponential rate dependence is directly expressed from the kinetic parameters of the reaction networks. We have also incorporated another effect of forming multimeric complexes in the method, the effect of having non-linear decay since more than one molecule may be removed at one instant, e.g. by spontaneous decay of a multimer, or by a multimer being consumed in some biochemical pathway. Since the time scale of the stability may change orders of magnitude between feedback from dimer binding and cooperative binding with otherwise similar reaction parameters we conclude that to understand phenotypic switching we need to be careful when formulating a model of an existing reaction network.

## Methods

### Escape rate calculation

Assume that we have a chemical species whose concentration is described by the variable *x*. Assume furthermore that the species are displaying two stable concentrations *x*_*L*_ and *x*_*H*_, a low and high stable concentration, respectively. The two stable concentrations are separated by the middle unstable concentration *x*_*M*_. We want to calculate the intrinsic stability of the two stable phenotypic states *x*_*L*_ and *x*_*H*_, that is, for how long will the system stay (on average) in a stable state before exit to the other stable state due to the intrinsic fluctuations generated from synthesis and decay of molecules. Individual trajectories *x*(*t*) evolve according to the stochastic differential equation


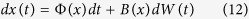


The term Φ(*x*) gives the average drift in concentration and *B*(*x*) the fluctuations, where *W*(*t*) is a Wiener Process. Notably, both the drift and the fluctuations depend on the concentration since both the drift and fluctuations are generated from the biochemical reactions of the system (which typically depends on the concentrations). Since the system is time-homogenous, i.e. the conditional probabilities satisfying Π(*x*′, *t* | *x*, 0) = Π(*x*′, 0 | *x*, −*t*), the backward Kolmogorov (or Fokker-Planck) equation corresponding to the stochastic process described in equation 12 can be written


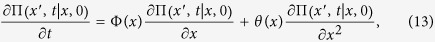


where 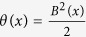. The probability that the concentration remains in the region **R** at time *t* starting from some concentration *x* inside **R** is 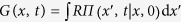. By integration of the backward equation 13 over *x*′ we get


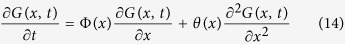


Now, the average time of escaping **R** starting in *x* is given by 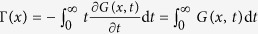, is therefore obeying the equation


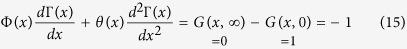


Introducing the quasi-potential Ψ(*x*) where 
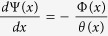
, gives the solution by the integrating factor *e*^−Ψ^. The escape time from the low state to the high state, Γ_*LH*_, is obtained by integrating twice, from 0 to *x*′ with boundary condition 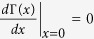, and from the low stable concentration, *x*_*L*_, to the high stable concentration, *x*_*H*_, with boundary condition Γ(*x*_*H*_) = 0 gives 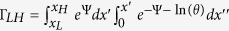. In the same manner we obtain the escape time from the high state to the low state, Γ_*HL*_. Integrating from ∞ to *x*′ with boundary condition 
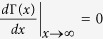
, and from *x*_*H*_ to *x*_*L*_ with boundary condition Γ(*x*_*L*_) = 0 gives 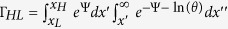. In these equations for the escape time, the external integrands get their main contribution near the middle unstable stationary point, *x*_*M*_, and the internal integrands get their main contribution near the low stable stationary point *x*_*L*_ for Γ_*LH*_, and near high stable stationary point *x*_*H*_ for Γ_*HL*_. Therefore, a parabolic approximation of the integrand functions around the dominant points gives the average escape time as


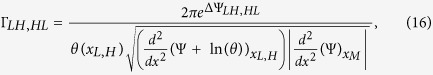


where ΔΨ_*LH*,*HL*_ = Ψ(*x*_*M*_) − Ψ(*x*_*L*,*H*_).

### System size scaling

Since we are interested in analysing the intrinsic, finite-molecule, stochastic properties of the bistable system it is convenient if the reaction network can be scaled from a system of few molecules to a large number of molecules without affecting the bi-stability. This can be achieved by introducing a system size parameter Ω^36^. In this setting we can view the scaled variables as concentrations, e.g. the protein monomer concentration *p* = *P*/Ω, such that all fixed point concentrations *x* = *X*/Ω are invariant under a change in Ω. Even though changing Ω can be viewed as a change in volume it should primarily be viewed as a scaling parameter that gives us additional freedom when choosing the number of molecules in the stable states.

### Fluctuation dissipation relation

For a multivariate Ornstein-Uhlenbeck process defined by the stochastic differential equation *d**x***(*t*) = −***A**x*(*t*)*dt* + ***B**dW*(*t*), where ***A*** and ***B*** are constant matrices, the covariance matrix ***C*** at stationary conditions satisfies a fluctuation-dissipation relation


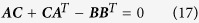


If ***x*** is sufficiently close to a stationary point ***x***_*s*_ we can for a more general system *d**x***(*t*) = −***f***(***x***(*t*))*dt* + ***B**dW*(*t*) linearise the drift ***f***(***x***(*t*)). The matrix ***A*** is then the Jacobian matrix evaluated at the stationary point ***x***_*s*_, 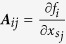. For a reaction network, the diffusion matrix is given by 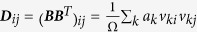, where *a*_*k*_ is the propensity of reaction *k* and *v*_*ki*_ the stoichiometry of species *i* in reaction *k*, evaluated at the stationary concentration ***x***_*s*_^36^. Once ***A*** and ***D*** are calculated we can compute the covariance matrix ***C*** through the fluctuation dissipation theorem 17. For a single variable process the variance is 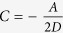.

## Additional Information

**How to cite this article**: Besya, A.-B. and Grönlund, A. Intrinsic phenotypic stability of a bi-stable auto regulatory gene. *Sci. Rep.* **6**, 22951; doi: 10.1038/srep22951 (2016).

## Supplementary Material

Supplementary Information
