# Supplementary material for: Intrinsic phenotypic stability of a bi-stable auto regulatory gene
Source: Sci Rep. 2016 Mar 10;6:22951. doi: 10.1038/srep22951 (PMC4785359; doi:10.1038/srep22951)
Supplement: Supplementary Information [file srep22951-s1.pdf]

Supplementary Information

Intrinsic phenotypic stability of a bi-stable auto regulatory gene

Azim-Berdy Besya and Andreas Grönlund*

*andreas.gronlund@umu.se

1 Chemical reaction network

We will investigate the two minimal reaction networks needed to generate bi-stability, positive transcriptional auto regulatory feedback; when the feedback are mediated through two proteins that form a complex on the promoter, and secondly when the proteins form dimers in the solution and then bind on the promoter. The participating molecules and their numbers are mRNA, M, free protein monomers, P, and protein dimers (in case of dimer formation), P_2. Each reaction k, its propensity a_k and stoichiometric coefficient $\boldsymbol{\nu}_k = (\Delta M, \Delta P, \Delta P_2)_k$ is listed, where ΔX are the number of molecules gained, or loss, in the specific reaction. The properties of a bistable system are highly dependent upon the details of the reaction rates e.g. synthesis and decay rates. Since we are interested in analysing the intrinsic stochastic properties of the bistable system it is convenient if the reaction network can be scaled from a system of few molecules to a large number of molecules without affecting the bi-stability. This can be achieved by introducing a system size parameter Ω (ref [22] in the main text). In this setting we can view the scaled variables as concentrations, e.g. the protein monomer concentration $p = P/\Omega$, such that all fixed point concentrations $x = X/\Omega$ are invariant under a change in Ω. Even though changing Ω can be viewed as a change in volume it should primarily be viewed as a scaling parameter that gives us additional freedom when choosing the number of molecules in the stable states. We have for both feedback processes assumed fast binding and unbinding of the proteins on the promoter such that the transcription rate can be approximated by the average binding occupancy of proteins on the promoter.

1.1 Cooperative binding feedback

Below we have listed the biochemical steps leading to positive autoregulation where two proteins bind cooperatively on two sites located at the promoter to assist RNA polymerase to bind and initiate transcription.

$$\begin{aligned}
&\text{Transcription,} && M \to M+1\,, && a_1 = \Omega F(p)\,, && \boldsymbol{\nu}_1 = \begin{bmatrix}1\\0\end{bmatrix}\\
&\text{Translation,} && P \to P+1\,, && a_2 = k_p M\,, && \boldsymbol{\nu}_2 = \begin{bmatrix}0\\1\end{bmatrix}\\
&\text{mRNA decay,} && M \to M-1\,, && a_3 = \gamma_m M\,, && \boldsymbol{\nu}_3 = \begin{bmatrix}-1\\0\end{bmatrix}\\
&\text{Protein decay,} && P \to P-1\,, && a_4 = \gamma_p P\,, && \boldsymbol{\nu}_4 = \begin{bmatrix}0\\-1\end{bmatrix}\\
&\text{The function } F(p) = k_{m,0} + k_{m,a}\frac{p^2}{K^2+p^2}
\end{aligned} \tag{S1}$$

The transcription rate of the free promoter is $k_{m,0}$ and the transcription rate when two proteins are present to assist the RNA polymerase to bind is $k_{m,0} + k_{m,a}$. The function $F(p)$ is the average occupancy of the protein complex on the promoter as a function of the protein concentration $p = P/\Omega$. We have assumed that the cooperative binding is strong and that both proteins are needed to assist the RNA polymerase to bind on the promoter, otherwise a linear term in p should be present both in the denominator and numerator of $F(p)$. K is the dissociation constant of the protein complex to its binding site on the promoter and gives the inverse strength of the binding.

1.2 Dimer binding feedback

Below we have listed the biochemical steps giving positive autoregulation by protein dimers binding on the promoter. Two proteins form a dimer in solution and can then bind at the promoter to assist RNA polymerase to bind and initiate transcription.

$$
\begin{array}{llll}
\text{Transcription,} & M \to M+1\,, & a_1 = \Omega F(p_2)\,, & \boldsymbol{v}_1 = \begin{bmatrix}1\\0\\0\end{bmatrix} \\
\text{Translation,} & P \to P+1\,, & a_2 = k_p M\,, & \boldsymbol{v}_2 = \begin{bmatrix}0\\1\\0\end{bmatrix} \\
\text{Dimerisation,} & P+P \to P_2\,, & a_3 = \frac{k_1}{\Omega}P^2\,, & \boldsymbol{v}_3 = \begin{bmatrix}0\\-2\\1\end{bmatrix} \\
\text{Un-dimerisation,} & P_2 \to P+P\,, & a_4 = k_{-1}P_2\,, & \boldsymbol{v}_4 = \begin{bmatrix}0\\2\\-1\end{bmatrix} \\
\text{mRNA decay,} & M \to M-1\,, & a_5 = \gamma_m M\,, & \boldsymbol{v}_5 = \begin{bmatrix}-1\\0\\0\end{bmatrix} \\
\text{Monomer decay,} & P \to P-1\,, & a_6 = \gamma_p P\,, & \boldsymbol{v}_6 = \begin{bmatrix}0\\-1\\0\end{bmatrix} \\
\text{Dimer decay,} & P_2 \to P_2-1\,, & a_7 = \gamma_{p_2} P_2\,, & \boldsymbol{v}_7 = \begin{bmatrix}0\\0\\-1\end{bmatrix}
\end{array}
\tag{S2}
$$

The function $F(p_2) = k_{m,0} + k_{m,a}\dfrac{p_2}{K_2+p_2}$

where $k_{m,0}$ is transcription rate of the free promoter and the transcription rate when a protein dimer is present to assist the RNA polymerase to bind is $k_{m,0}+k_{m,a}$. The dissociation constant K_2 gives the (inverse) binding strength of dimers on the promoter and the function $F(p_2)$ is the average occupancy of dimers on the promoter as a function of the number of dimers in solution.

Most calculations and derivations that will follow are performed for dimer mediated feedback since the results for the cooperative promoter feedback can be obtained as a special case by the right choice of parameters. Stochastic simulations of the above two type of feedback systems, cooperative binding feedback and dimer binding feedback, are implemented as a reference for the analytical derivations.

2 Large molecule number limit calculations

In the large molecule limit, $\Omega \to \infty$, the concentrations $(m,p,p_2) = (M,P,P_2)/\Omega$ become continuous and reactions occur continuous in time. The concentrations of the molecules in equation S2 will in the large molecule limit evolve according to the following set of ordinary differential equations (ODE)

$$
\begin{aligned}
\frac{dm}{dt} &= F(p_2) - \gamma_m m \\
\frac{dp}{dt} &= k_p m + 2k_{-1}p_2 - 2k_1 p^2 - \gamma_p p \\
\frac{dp_2}{dt} &= k_1 p^2 - k_{-1}p_2 - \gamma_{p_2} p_2
\end{aligned}
\tag{S3}
$$

When mRNA turnover is fast compared to other reactions, $\frac{dm}{dt} = 0$, we have that $m = F(p_2)/\gamma_m$. Then we have from equation S3 for the total concentration $p_T = p + 2p_2$,

$$
\frac{dp_T}{dt} = \frac{d(p+2p_2)}{dt} = F(p_2) - \gamma_p p - 2\gamma_{p_2} p_2 \tag{S4}
$$

If monomers and dimers are (rapidly) partitioned according to the dissociation constant $K_1 = k_{-1}/k_1$, then $p_2 = p^2/K_1$. We can now formulate a closed form differential equation for *any* of the concentrations p, p_2 or p_T since each can be expressed

from the other two. For the bifurcation analysis that follows it is most convenient to write the equation in terms of a scaled monomer concentration $\rho = \frac{p}{K}$, with $K^2 = K_1K_2$ and scaled time $\tau = \gamma_p t$

$$\begin{aligned}
\frac{d\rho_T}{d\tau} &= \frac{d}{d\tau}(\rho + 2\rho_2) = \frac{d}{d\tau}\left(\rho + \frac{2K}{K_1}\rho^2\right) = \left(1 + \frac{4K}{K_1}\rho\right)\frac{d\rho}{d\tau} = S_0 + S_a\left(\frac{\rho^2}{1+\rho^2}\right) - (\rho + \alpha\rho^2) \equiv \Phi_{\rho_T,\mathrm{DIM}}(\rho) \\
\Rightarrow \frac{d\rho}{d\tau} &= \left(1 + \frac{4K}{K_1}\rho\right)^{-1}\left(S_0 + S_a\left(\frac{\rho^2}{1+\rho^2}\right) - (\rho + \alpha\rho^2)\right) \equiv \Phi_{\rho,\mathrm{DIM}}(\rho)
\end{aligned} \tag{S5}$$

where $S_0 = \frac{k_0}{K}\frac{k_p}{\gamma_m\gamma_p}$, $S_a = \frac{k_a}{K}\frac{k_p}{\gamma_m\gamma_p}$, $\rho = \frac{p}{K}$ and $\alpha = 2\frac{\gamma_{P_2}}{\gamma_P}\frac{K}{K_1}$. The subscript ρ and ρ_T for the function Φ are denoting whether the function generates the dynamics of the monomer concentration ρ or the total concentration ρ_T for dimer binding feedback. The two descriptions are equivalent since all concentrations are uniquely determined, e.g. knowing ρ uniquely gives ρ_T and ρ_2. Similar calculations as above for the cooperative binding feedback gives that

$$\frac{d\rho}{d\tau} = S_0 + S_a\left(\frac{\rho^2}{1+\rho^2}\right) - \rho \equiv \Phi_{\mathrm{CP}}(\rho) \tag{S6}$$

We can see that this result is also obtained by letting $K_1 \to \infty$ (i.e. no dimers) which gives $\alpha = 0$ in equation S5. Note that for the case of dimers mediating the feedback – no dimers would result in no feedback. However, the operation can be performed to obtain the result for cooperative binding feedback, even though feedback is then physically mediated by monomers. We will use the result later as a quick route of deriving results for cooperative binding feedback.

2.1 Stability and bifurcation analysis

Assume that we have a potential function $U(x)$ such that a change in the concentration x is driven by the drift term, $\Phi(x) = -\frac{dU}{dx}$. For the dimer binding feedback the drift (as a function on monomer proteins) equals the RHS of equation S5, $\Phi_{\rho.\mathrm{DIM}}(\rho)$ or equivalently $\Phi_{\rho_T.\mathrm{DIM}}(\rho)$. For cooperative binding feedback the drift equals the RHS of equation S6, $\Phi_{\mathrm{CP}}(\rho)$. The observed concentration will change until the system is resting at a minima in $U(x)$ where its derivative is zero and its second derivative positive. This is called a *stable* steady state. An *unstable* steady state is where $U(x)$ is having a maximum. For the rate parameters where the system displays bi-stability there are always two stable stationary solutions separated by an unstable one and the region of bi-stability is given by the interval $(\rho_{*,1}, \rho_{*,2})$ where both endpoints are solutions of $\Phi(\rho_*) = 0$ and $\Phi'(\rho_*) = 0$. The region of bi-stability is given by

$$\begin{aligned}
S_a(\rho_*) &= \frac{(1+2\alpha\rho_*)(1+\rho_*^2)^2}{2\rho_*} \\
S_0(\rho_*) &= \rho_* + \alpha\rho_*^2 - \frac{\rho_*}{2}(1+2\alpha\rho_*)(1+\rho_*^2),
\end{aligned} \tag{S7}$$

where ρ_* are the monomer concentrations separating the bi-stable region. The region of bi-stability in (S_a, S_0) space can for each choice of α be visualised by plotting the parametric curves of S_0 and S_a against ρ_*. Moreover, it does not matter if we express the RHS of equation S7 in terms of monomer, dimer or total concentration – the region of bi-stability in (S_a, S_0) space is the same. The bifurcation for the cooperative binding feedback is obtained by letting $\alpha = 0$.

3 Finite molecule number calculations and intrinsic stability

In the large molecule limit, where reactions are assumed to become continuous, the concentrations will converge to a (stable) stationary solution of the differential equations S3 (and equation S5) and stay there, unless the rate constants will change and another stationary solution is assumed. However, since the number of molecules are finite, the time between events of synthesis and decay will not be continuous but random and exponentially distributed with mean following the reaction propensities. The deviation from the stationary number of molecules that each reaction results in are also related to the total number of molecules since the smallest state jump is of the order of one molecule. Thus, for all systems consisting of a finite number of molecules we need to have a description of the noise and deviation from the stationary concentration. Such a description can be expressed differently, e.g. via a chemical master equation describing the probability of the different chemical states in time, or (as here) translating the chemical reaction system to a stochastic differential equation (SDE). We will use the former route since we are interested in calculating the escape rate from the stable states to compute their intrinsic stability. Moreover, we can express the stochastic properties of the system using any of the concentrations ρ, ρ_2 or ρ_T as long as both the drift and noise term are describing the noise of the same quantity. The part describing the non-stochastic contribution to the evolution of the total concentration we already have, which is given by the right hand side of equation S5 (with $\alpha = 0$ for cooperative binding

feedback). The stochastic contribution to the SDE we have to calculate, but before we do so we start with formulating the SDE and its corresponding Fokker-Planck equation (for the total protein concentration),

$$
\begin{aligned}
d\rho_T(\tau) &= \Phi(\rho_T)d\tau + B(\rho_T)dW(\tau) \\
\frac{\partial \Pi(\rho_T,\tau)}{\partial \tau} &= \Phi(\rho_T)\frac{\partial\left(\Pi(\rho_T,\tau)\right)}{\partial \rho_T} + \theta(\rho_T)\frac{\partial\left(\Pi(\rho_T,\tau)\right)}{2\partial \rho_T^2}
\end{aligned}
\tag{S8}
$$

Where $\Pi(\rho_T,\tau)$ is the probability distribution of the scaled total protein concentration, the drift term $\Phi(\rho_T)$ the right hand side of equation , and $W(\tau)$ is a Wiener Process. Now we need to quantify the diffusive noise term $\theta(\rho_T) = \frac{B^2(\rho_T)}{2}$. For a multivariate Ornstein-Uhlenbeck process defined by the stochastic differential equation $dx(t) = -\boldsymbol{A}x(t)dt + \boldsymbol{B}dW(t)$, where $\boldsymbol{A}$ and $\boldsymbol{B}$ are constant matrices, the covariance matrix $\boldsymbol{C}$ at stationary conditions satisfies a fluctuation-dissipation relation

$$
\boldsymbol{AC} + \boldsymbol{CA}^T - \boldsymbol{BB}^T = 0 \tag{S9}
$$

If x is sufficiently close to a stationary point x_s we can for a more general system linearise the equations, e.g. S3, such that the fluctuation-dissipation relation S9 (approximately) holds. The matrix $\boldsymbol{A}$ is then the Jacobian matrix evaluated at the stationary point x_s, and if we express the reactions in the two coordinates mRNA, m, and total protein, p_T, we obtain

$$
\mathbf{A} = \begin{bmatrix} \frac{\partial f_m}{\partial m} & \frac{\partial f_m}{\partial p_T} \\ \frac{\partial f_p}{\partial m} & \frac{\partial f_p}{\partial p_T} \end{bmatrix}_{(m,p_T)_S}, \tag{S10}
$$

where $f_m = F(p_2) - \gamma_m m$ and $f_p = k_p m - (\gamma_p p + 2\gamma_{p_2} p_2)$ which gives that

$$
\mathbf{A} = \begin{bmatrix} -\gamma_m & \frac{\partial F(p_2)}{\partial p_T} \\ k_p & -\gamma_P(1+2\beta\frac{\partial p_2}{\partial p_T}) \end{bmatrix}_{(m,p_T)_S}, \tag{S11}
$$

where $\beta = \frac{\gamma_{p_2}}{\gamma_p} - 1$. The diffusion matrix $\boldsymbol{D}_{ij} = (\boldsymbol{BB}^T)_{ij} = \frac{1}{\Omega}\sum_k a_k v_{ki} v_{kj}$ for this set of reactions in stationary conditions $f_m = 0$ and $f_p = 0$ is

$$
\mathbf{D} = \frac{1}{\Omega}\begin{bmatrix} 2\gamma_m m & 0 \\ 0 & 2\gamma_P(p_T + (3\beta+1)p_2) \end{bmatrix}_{(m,p_T)_S}, \tag{S12}
$$

The covariance matrix $\boldsymbol{C}$ can now be calculated from the fluctuation dissipation equation S9, which gives the variance of the total concentration of proteins at a stationary point as

$$
\mathbf{C}_{22} = \left[\frac{1}{\Omega}\frac{k_p}{\gamma_m + \gamma_p(1+2\beta\frac{\partial p_2}{\partial p_T})} \frac{\frac{\frac{\partial F(p_2)}{\partial p_T}(p_T+(3\beta+1)p_2)}{\gamma_p(1+2\beta\frac{\partial p_2}{\partial p_T})} + p_T + 2\beta p_2}{(1+2\beta\frac{\partial P_2}{\partial p_T}) - \frac{F' k_p}{\gamma_p\gamma_m}} + \frac{1}{\Omega}\frac{p_T + (3\beta+1)p_2}{1+2\beta\frac{\partial p_2}{\partial p_T}} \right]_{(p,p_2,p_T)_S} \tag{S13}
$$

Now, assuming that mRNA turnover and protein dimerization are fast compared to the other time scales in the system we will collapse the reaction system listed in equations S2 with only synthesis and decay of proteins and its impact on the total number of proteins. One species, P_T, and three reactions,

$$
\begin{array}{lll}
\text{Synthesis, } P_T \to P_T + q\,, & a_1 = \frac{k_p}{\gamma_m}\frac{\Omega F(p_T)}{q}\,, & v_1 = q \\
\text{Decay of monomers, } P_T \to P_T - 1\,, & a_2 = \gamma_p P\,, & v_2 = -1 \\
\text{Decay of dimers, } P_T \to P_T - 2\,, & a_3 = \gamma_{p_2} P_2\,, & v_3 = -2
\end{array}
\tag{S14}
$$

where q is number of total proteins added, i.e. the stoichiometry, per synthesis is now including both transcription and translation. We can think of q as a parameter that accounts for the fact that the intrinsic stochasticity of protein synthesis propagates from transcription to translation and, if mRNA is short lived and translation fast, the multiple proteins translated per mRNA can be viewed as a stoichiometric effect on the synthesis events appearing with the rate of transcription initiation. If we want to collapse the reactions of equation S2 with the reduced set of reactions listed in S14 and recover the stochastic part we should

match the covariance of the total concentration of proteins. For a scalar system the fluctuation dissipation relation, equation S9, becomes an algebraic equation and the variance is given by $C = -D/2A$ with

$$A = \left[\frac{k_p}{\gamma_m}\frac{dF(p_T)}{dp_T} - \gamma_p(1+2\beta\frac{\partial p_2}{\partial p_T})\right]_{(p,p_2,p_T)_S} \tag{S15}$$

and the diffusive component

$$D = \left[\frac{\gamma_p}{\Omega}\left(2(p_T+(3\beta+1)p_2)+(q-1)(p_T+2\beta p_2)\right)\right]_{(p,p_2,p_T)_S} \tag{S16}$$

Now, setting $C = \mathbf{C}_{22}$, gives q as

$$q = \left(1+2\frac{k_p}{\gamma_m+\gamma_p(1+2\beta\frac{\partial p_2}{\partial p_T})}\right) + \frac{2\frac{\partial F(p)}{\partial p_T}k_p(p_T+(3\beta+1)p_2)}{\gamma_p(1+2\beta\frac{\partial p_2}{\partial p_T})(p_T+2\beta p_2)}\left(\frac{1}{\gamma_m+\gamma_p(1+2\beta\frac{\partial p_2}{\partial p_T})} - \frac{1}{\gamma_m}\right) \tag{S17}$$

When $\gamma_m \gg \gamma_p(1+2\beta\frac{\partial p_2}{\partial p_T})$ we obtain $q = 1+2\frac{k_p}{\gamma_m}$. For the stochastic differential equation and its Fokker-Planck equation S8 describing the scaled total concentration $\rho_T = p_T/K$ we obtain the noise term $B(\rho_T)$ by combining equations S16 and S17

$$\theta(\rho_T) = \frac{B^2(\rho_T)}{2} = \frac{B^2(p_T)}{2K^2\gamma_p} = \frac{D}{2K^2\gamma_p} = \frac{1}{K\Omega}\left(\left(1+\frac{k_p}{\gamma_m}\right)\rho_T + \left(\frac{\gamma_{p_2}}{\gamma_p}+2\beta\left(1+\frac{k_p}{\gamma_m}\right)\right)\rho_2\right) \tag{S18}$$

Now, the term $B(\rho_T)$ (and $\theta(\rho_T)$) is strictly speaking only valid at a stable stationary point, $(p,p_2,p_T)_S$ i.e the two stable points of a bi-stable system, since we have done the calculations under such assumptions. However, we approximate $B(\rho_T)$ to have the same dependence on ρ_T for all non-stationary points as well.
For the dimer binding feedback the SDE and its Focker-Planck equation can be expressed in closed form in terms of the total concentration, since $\rho_2 = \frac{K}{K_1}\left(\sqrt{\frac{K_1}{2K}\left(\rho_T+\frac{K_1}{8K}\right)} - \frac{K_1}{4K}\right)^2$,

$$\begin{aligned}
\Phi(\rho_T) &= \frac{d\rho_T}{d\tau} = S_0 + S_a\frac{\left(\sqrt{\frac{K_1}{2K}\left(\rho_T+\frac{K_1}{8K}\right)}-\frac{K_1}{4K}\right)^2}{1+\left(\sqrt{\frac{K_1}{2K}\left(\rho_T+\frac{K_1}{8K}\right)}-\frac{K_1}{4K}\right)^2} - \left(\sqrt{\frac{K_1}{2K}\left(\rho_T+\frac{K_1}{8K}\right)}-\frac{K_1}{4K}+\alpha\left(\sqrt{\frac{K_1}{2K}\left(\rho_T+\frac{K_1}{8K}\right)}-\frac{K_1}{4K}\right)^2\right)\\
\theta(\rho_T) &= \frac{1}{K\Omega}\left(\left(1+\frac{k_p}{\gamma_m}\right)\rho_T + \frac{K}{K_1}\left(\frac{\gamma_{p_2}}{\gamma_p}+2\beta\left(1+\frac{k_p}{\gamma_m}\right)\right)\left(\sqrt{\frac{K_1}{2K}\left(\rho_T+\frac{K_1}{8K}\right)}-\frac{K_1}{4K}\right)^2\right)
\end{aligned} \tag{S19}$$

For the cooperative binding feedback we have the drift $\Phi(\rho)$ is given by RHS of equation S6. The diffusion term $\theta(\rho)$ in the Fokker-Planck equation S8 is obtained by letting $K_1 \to \infty$, giving $\rho_T = \rho$ and $\rho_2 = 0$ in equation S18

$$\begin{aligned}
\Phi(\rho) &= \frac{d\rho}{d\tau} = S_0 + S_a\left(\frac{\rho^2}{1+\rho^2}\right) - \rho\\
\theta(\rho) &= \frac{1}{K\Omega}\left(1+\frac{k_p}{\gamma_m}\right)\rho
\end{aligned} \tag{S20}$$

4 Escape time calculation

Using Eq. S8 for probability distribution of the scaled total protein concentration, the probability of this variable that remains in region $\mathbf{R}$ at time τ, $G(\rho_T,\tau) = \int_{\mathbf{R}}\Pi(x,\tau\,|\,\rho_T,0)\mathrm{d}x$, obeys the equation

$$\frac{\partial G(\rho_T,\tau)}{\partial\tau} = \Phi(\rho_T)\frac{\partial G(\rho_T,\tau)}{\partial\rho_T} + \theta(\rho_T)\frac{\partial^2 G(\rho_T,\tau)}{\partial\rho_T^2} \tag{S21}$$

integrating Eq. S21 over time interval $(0,\infty)$ gives the differential equation governing escape time, $\Gamma(\rho_T) = -\int_0^\infty \frac{\partial G(\rho_T,\tau)}{\partial t} d\tau$,

$$\Phi(\rho_T)\frac{d\Gamma(\rho_T)}{d\rho_T} + \theta(\rho_T)\frac{d^2\Gamma(\rho_T)}{d\rho_T^2} = -1 \tag{S22}$$

Division of both side of this equation by $\theta(\rho_T)$, introduction of quasi-potential Ψ where $\frac{d\Psi(\rho_T)}{d\rho_T} = -\frac{\Phi(\rho_T)}{\theta(\rho_T)}$, and multiplication of both sides by $e^{-\Psi}$ gives $\frac{d}{d\rho_T}(e^{-\Psi}\frac{d\Gamma(\rho_T)}{d\rho_T}) = -e^{-\Psi-\ln(\theta)}$. Double integration of this equation from 0 to y with boundary condition $\frac{d\Gamma(y)}{dy}|_{y=0} = 0$, and from low concentration stable point, ρ_{T_L}, to high concentration stable point, ρ_{T_H}, with boundary condition $\Gamma(\rho_{T_H}) = 0$ gives escape time from the low state to the high state, $\Gamma_{LH} = \int\limits_{\rho_{T_L}}^{\rho_{T_H}} e^{\Psi} dy \int\limits_0^y e^{-\Psi-\ln(\theta)} dz$. Double integration from ∞ to y with boundary condition $\frac{d\Gamma(y)}{dy}|_{y\to\infty} = 0$, and from ρ_{T_H} to ρ_{T_L} with boundary condition $\Gamma(\rho_{T_L}) = 0$ gives the escape time from the high state to the low state, $\Gamma_{HL} = \int\limits_{\rho_{T_L}}^{\rho_{T_H}} e^{\Psi} dy \int\limits_y^\infty e^{-\Psi-\ln(\theta)} dz$. In these equations for the escape time, the external integrands get their main contribution near the middle unstable stationary point, ρ_{T_M}, and the internal integrands get their main contribution near the low stable stationary point ρ_{T_L} for Γ_{LH}, and near the high stable stationary point ρ_{T_H} for Γ_{HL}. Therefore, a parabolic approximation of the integrand functions around the dominant points,

$$\Gamma_{LH,HL} = \int\limits_{-\infty}^{\infty} e^{\Psi(\rho_{T_M})+\frac{1}{2}(y-\rho_{T_M})^2\frac{d^2}{d\rho_T^2}(\Psi)|_{\rho_{T_M}}} dy \int\limits_{-\infty}^{\infty} e^{-(\Psi+\ln(\theta))|_{\rho_{T_{L,H}}}-\frac{1}{2}(z-\rho_{T_{L,H}})^2\frac{d^2}{d\rho_T^2}(\Psi+\ln(\theta))|_{\rho_{T_{L,H}}}} dz \tag{S23}$$

gives the escape time (in real time after division by γ_p, since $t = \tau/\gamma_p$),

$$\Gamma_{LH,HL} = \frac{2\pi e^{\Delta\Psi_{LH,HL}}}{\gamma_P\theta(\rho_{T_{L,H}})\sqrt{\frac{d^2}{d\rho_T^2}(\Psi+\ln(\theta))|_{\rho_{T_{L,H}}}|\frac{d^2}{d\rho_T^2}(\Psi)|_{\rho_{T_M}}|}} \tag{S24}$$

where $\Delta\Psi_{LH,HL} = \Psi(\rho_{T_M}) - \Psi(\rho_{T_{L,H}})$, the quasi-potential for cooperative binding feedback is

$$\Psi(\rho) = \frac{K\Omega}{1+\frac{k_p}{\gamma_m}}\left(-S_0\ln(\rho) - \frac{S_a}{2}\ln(1+\rho^2) + \rho\right) \tag{S25}$$

and the quasi-potential for dimer binding feedback is

$$\Psi(\tilde{\rho}_T) = \frac{K\Omega}{1+\frac{k_p}{\gamma_m}}\left(\ln\left(\frac{(\sqrt{\tilde{\rho}_T}+\delta_1)^{n_1}(\sqrt{\tilde{\rho}_T}+\delta_2)^{n_2}}{\left(\left(\sqrt{\tilde{\rho}_T}-\sqrt{\frac{K_1}{8K}}\right)^2+\frac{1}{4}\left(\frac{K_1}{K}+\frac{8K}{K_1}\right)\right)^{n_3}}\right) + n_4\tan^{-1}\left(\frac{\sqrt{\tilde{\rho}_T}-\sqrt{\frac{K_1}{8K}}}{\frac{1}{2}\sqrt{\frac{K_1}{K}+\frac{8K}{K_1}}}\right) + n_5\sqrt{\tilde{\rho}_T} + n_6\tilde{\rho}_T\right) \tag{S26}$$

where $\tilde{\rho}_T = \rho_T + \frac{K_1}{8K}$, $\delta_1 = \frac{\xi_1}{2}\left(1+\sqrt{1-\frac{4\xi_2}{\xi_1^2}}\right)$, $\delta_2 = \frac{\xi_1}{2}\left(1-\sqrt{1-\frac{4\xi_2}{\xi_1^2}}\right)$, $n_1 = -\frac{S_0+S_a}{1+\frac{\xi_0}{2}}\left(1+\frac{1}{\sqrt{1-\frac{4\xi_2}{\xi_1^2}}}\right) + \frac{4S_a\xi_5K}{K_1\left(1+\frac{\xi_0}{2}\right)} + \frac{\alpha K_1}{2K\left(1+\frac{\xi_0}{2}\right)\sqrt{1-\frac{4\xi_2}{\xi_1^2}}}\left(\xi_2\left(\frac{1}{\alpha}\sqrt{\frac{2K}{K_1}}-\sqrt{\frac{K_1}{2K}}-\xi_1\right)+\delta_1\left(\frac{K_1}{8K}+\frac{1}{2\alpha}-\xi_2\right)\right)$, $n_2 = -\frac{S_0+S_a}{1+\frac{\xi_0}{2}}\left(1+\frac{1}{\sqrt{1-\frac{4\xi_2}{\xi_1^2}}}\right) + \frac{4S_a\xi_6K}{K_1\left(1+\frac{\xi_0}{2}\right)} + \frac{\alpha K_1}{2K\left(1+\frac{\xi_0}{2}\right)\sqrt{1-\frac{4\xi_2}{\xi_1^2}}}\left(-\xi_2\left(\frac{1}{\alpha}\sqrt{\frac{2K}{K_1}}-\sqrt{\frac{K_1}{2K}}-\xi_1\right)+\left(2\sqrt{1-\frac{4\xi_2}{\xi_1^2}}-\delta_1\right)\left(\frac{K_1}{8K}+\frac{1}{2\alpha}-\xi_2\right)\right)$, $n_3 = -\frac{2S_a\xi_3K}{K_1\left(1+\frac{\xi_0}{2}\right)}$,

$n_4 = \frac{4S_aK}{K_1\left(1+\frac{\xi_0}{2}\right)}\left(\xi_4+\xi_3\sqrt{\frac{K_1}{8K}}\right)$, $n_5 = \frac{\alpha K_1}{K\left(1+\frac{\xi_0}{2}\right)}\left(\frac{1}{\alpha}\sqrt{\frac{2K}{K_1}}-\sqrt{\frac{K_1}{2K}}-\xi_1\right)$, $n_6 = \frac{\alpha K_1}{K(2+\xi_0)}$, $\xi_0 = 2\beta+\frac{1}{1+b}\frac{\gamma_{p2}}{\gamma_p}$, $\xi_1 = -\frac{\sqrt{\frac{K_1}{2K}}}{1+\frac{2}{\xi_0}}$, $\xi_2 = \frac{K_1}{8K}\left(1-\frac{2}{1+\frac{\xi_0}{2}}\right)$ and

$$\begin{pmatrix}\xi_3\\ \xi_4\\ \xi_5\\ \xi_6\end{pmatrix} = \begin{pmatrix} 1 & 0 & 1 & 1 \\ \delta_1+\delta_2 & 1 & -\sqrt{\frac{K_1}{2K}}+\delta_2 & -\sqrt{\frac{K_1}{2K}}+\delta_1 \\ \delta_1\delta_2 & \delta_1+\delta_2 & \frac{3K_1}{8K}+\frac{2K}{K_1}-2\delta_2\sqrt{\frac{K_1}{8K}} & \frac{3K_1}{8K}+\frac{2K}{K_1}-2\delta_1\sqrt{\frac{K_1}{8K}} \\ 0 & \delta_1\delta_2 & \delta_2\left(\frac{3K_1}{8K}+\frac{2K}{K_1}\right) & \delta_1\left(\frac{3K_1}{8K}+\frac{2K}{K_1}\right)\end{pmatrix}^{-1}\begin{pmatrix}0\\0\\1\\0\end{pmatrix}.$$

In the constant noise approximation when the fluctuations are set to be $\theta(\rho_{T_{L,H}})$, evaluated at the stable points L and H, for dimer binding, the quasi potential would be

$$\Psi(\rho) = \frac{K\Omega}{1+\frac{k_p}{\gamma_m}}\left(\sum_{i=1}^{4} n_i\rho^i + n_5\tan^{-1}(\rho)+n_6\ln(1+\rho^2)\right) \tag{S27}$$

where $n_1 = -\frac{S_0+S_a}{\xi}$, $n_2 = \frac{1}{2\xi}-\frac{2K}{K_1\xi}(S_0+S_a)$, $n_3 = \frac{1}{3\xi}\left(\frac{4K}{K_1}+\alpha\right)$, $n_4 = \frac{K\alpha}{K_1\xi}$, $n_5 = \frac{S_a}{\xi}$, $n_6 = \frac{2KS_a}{K_1\xi}$ and

$$\xi = \rho_{T_{L,H}}+\frac{K}{K_1}\left(2\beta+\frac{\gamma_{p2}}{\gamma_p}\frac{1}{1+\frac{k_p}{\gamma_m}}\right)\left(\sqrt{\frac{K_1}{2K}\left(\rho_{T_{L,H}}+\frac{K_1}{8K}\right)}-\frac{K_1}{4K}\right)^2,$$

For cooperative binding, the quasi potential is in the constant noise approximation is (again with $\theta(\rho_{T_{L,H}})$, evaluated at the stable points L and H)

$$\Psi(\rho) = \frac{K\Omega}{1+\frac{k_p}{\gamma_m}}\frac{1}{\rho_{L,H}}\left(-(S_0+S_a)\rho+\frac{1}{2}\rho^2+S_a\tan^{-1}(\rho)\right) \tag{S28}$$

5 Integration of the pseudo potential

Here we will give the main steps when integrating the pseudopotential, whic is given by $\Psi = \int \frac{\Phi(\rho_T)}{\theta(\rho_T)}d\rho_T$. The monomer and dimer concentration can be written in the variable $\tilde{\rho}_T = \rho_T + K_1/8K$. Now,

$$\rho = A_0\sqrt{\tilde{\rho}}_T + B_0 \tag{S29}$$

with $A_0 = \sqrt{K_1/2K}$ and $B_0 = -K_1/4K$, and

$$\rho_2 = \frac{K}{K_1}\rho^2 = C_1(\tilde{\rho}_T + A_1\sqrt{\tilde{\rho}_T}+B_1) \tag{S30}$$

with $C_1 = K_1/2K$, $B_1 = K_1/8K$ and $A_1 = -\sqrt{K_1/2K}$. With this substitution the noise can be written in the form

$$\theta(\tilde{\rho}_T) = C_2(\sqrt{\tilde{\rho}_T}+\delta_1)(\sqrt{\tilde{\rho}_T}+\delta_2) \tag{S31}$$

where C_2 can be determined from the parameters. Expressing the concentration drift, $\Phi(\tilde{\rho}_T)$ in the variable $\tilde{\rho}_T$ and then making the substitution $z = \sqrt{\tilde{\rho}_T}$ giving $d\tilde{\rho}_T = 2zdz$ gives that the pseudo-potential can be integrated by elementary functions.
